# Reflections on timing of motherhood - a qualitative online study with women of reproductive age

**DOI:** 10.1186/s12905-024-03409-0

**Published:** 2024-11-05

**Authors:** Camilla Gry Temmesen, Henriette Svarre Nielsen, Kathrine Birch Petersen, Jane Clemensen

**Affiliations:** 1 Department of Health & Rehabilitation, University College Absalon, Slagelse, Denmark; 2 Institute of Clinical Research, Faculty of Health Sciences, University of Southern Denmark, Odense, Denmark; 3 Centre for Innovative Medical Technology, Odense University Hospital, Odense, Denmark; 4 HCA Research, Hans Christian Andersen Children’s Hospital, Odense University Hospital, Odense, Denmark; 5 Dept. of Obstetrics and Gynecology, Copenhagen University Hospital, Hvidovre, Denmark; 6 Dept. of Clinical Medicine, University of Copenhagen, Copenhagen, Denmark; 7 Birch Gynecology and Fertility, Copenhagen, Copenhagen, Denmark

**Keywords:** Timing, Postponing, Reproduction, Motherhood, Women, Reproductive age, Facebook, Online focus groups, Social media

## Abstract

**Background:**

The age of Danish women giving birth to their first child has risen throughout the last 50 years, and the number of women and men who are having their first child when they are at advanced maternal or paternal age is increasing worldwide. Postponing parenthood is not unique to Denmark, as the same pattern is seen especially in other European and Western countries. The aim of this study was in a social media setting to explore reflections on the timing of motherhood among Danish women of reproductive age who not yet had children.

**Method:**

This study was a qualitative study based on three online focus groups on Facebook. Twenty-six women of reproductive age discussed the timing of motherhood for three days in January 2020. Data were analyzed using Systematic Text Condensation.

**Results:**

Three main themes were identified: ‘*Life before parenthood’*, *‘To plan for a child’* and *‘A life without children’.* Several external and internal conditions influence whether and if so *when* women consider becoming mothers. Regarding the biological capacity for having children, women compare themselves with their female relatives and friends and colleagues regarding the social norms surrounding motherhood. Women with no children often experience either positive or negative pressure from family, friends, and colleagues regarding when to have children.

**Conclusion:**

Several internal and external considerations influence *when* and *if* women become mothers. Social surroundings such as family, friends, and colleagues have an impact on women’s reproductive considerations in terms of pressure to have children in the future. Danish women tend to compare their reproductive capacity to their female relatives and compare the social norms surrounding motherhood to their female friends. Women in this study were positive towards discussing the timing of motherhood with other women of reproductive age on social media.

**Supplementary Information:**

The online version contains supplementary material available at 10.1186/s12905-024-03409-0.

## Abstract (plain language summary)

The age of women giving birth to their first child has risen throughout the last 50 years, and the number of Danish women and men having their first child when they are at advanced maternal or paternal is increasing. Postponing motherhood is not unique to Denmark, as the same pattern is seen especially in other European and Western countries. In this study, we explored the reflections on the timing of motherhood among Danish women of reproductive age who had not yet had children. Using a novel approach, we conducted three online focus groups on the social media platform Facebook. Twenty-six women aged 18–43 years without children, regardless of whether they wanted to have children in the future, were included. The study revealed several internal and external considerations influencing *when* and *if* women establish a family. Regarding their biological capacity for having children, women tend to compare themselves to their female relatives and their friends and colleagues regarding the social norms surrounding motherhood. Women with no children often experience pressure from family, friends, and colleagues about when to have children, which can be perceived as unfavorable while at the same time being experienced as motivating for planning future motherhood. Women in this study were positive towards discussing the timing of motherhood with other women of reproductive age on social media.

**Supplementary Information:**

The online version contains supplementary material available at 10.1186/s12905-024-03409-0.

## Introduction

Postponing motherhood is a general global trend especially in European and Western countries [1–3]. In 1970, the average age of Danish women delivering their first child was 23.7 years and has increased steadily over the past decades to 30.0 years in 2023 [4]. The same increase is seen for Danish first-time fathers, who in general are slightly older than the women, with an average of 31.7 years of age when they have their first child [4]. This tendency of postponing parenthood is not solely present in Denmark. It is also reported by other European and Western countries such as the USA and Korea and is seemingly an increasing global health challenge [5–9]. As a consequence of the general increase in the rising age trend in the timing of parenthood, the number of Danish women giving birth to their first child in their mid- or late thirties or above has doubled, rising from 6.7% in 1998 to 12.3% in 2015 [10], an age that in most studies is described as being at ‘advanced maternal age’ (AMA ³ 35 years). Becoming a mother above the age of 35 years is problematic, as it increases the risk of infertility and pregnancy- and birth complications, such as miscarriages [11, 12], chromosomal abnormalities [3, 11], hypertensive complications, fetal growth restrictions and fetal death [3, 13], as well as an increased risk of having a cesarean section [10]. Though male age is less significant, it is not without implications for the male reproductive abilities. Above 40 years, which is the definition of ‘advanced paternal age’ (APA ³ 40 years), there is an increased risk of lower sperm quality just as there is a greater risk of infertility, a longer time-to-pregnancy, a higher prevalence of genetic disorders (especially achondroplasia) and a higher risk of spontaneous abortions and stillbirths [11, 14]. In addition, a higher age causes an increased risk of other risk factors, such as hypertension, diabetes, cancer, sexually transmitted diseases, overweight, and a higher risk of long-term exposure to hormonal disruptive chemicals that may affect male and female fertility [11, 15, 16].

Schmidt et al. (2011) found that highly educated women who risk losing income or damaging their work career when becoming mothers tend to postpone motherhood. The probability of having a first or second child after the age of 35 years increases when highly educated. In addition, there is a lower chance of couples reaching their desired family size when parenthood is postponed [15]. In a meta-synthesis, Temmesen et al. (2023) identified that although some women express concern about whether it could become complicated to achieve pregnancy if postponing motherhood, women do not express much concern about the medical risks of age-related pregnancy- and birth complications [1].

Several studies highlight that both men and women of reproductive age lack fertility knowledge, especially regarding the age-related fertility decline [17–19]. The International Committee for Monitoring Assisted Reproductive Technologies (ICMART) defines fertility knowledge or *fertility awareness* as the understanding of reproductive matters, including the understanding of individual, non-individual and other risk factors, such as advanced age, sexually transmitted infections, smoking, obesity, and societal and cultural factors affecting family planning [20]. In a review, Pedro et al. (2018) found low to moderate fertility awareness among people of reproductive age, accordingly a lack of awareness of the age-related infertility decline and a tendency to overestimate the chance of successful pregnancy by assisted reproductive technologies [17]. This is supported by a review by Ren et al. (2023), who explored fertility awareness among university students and found female and medical students to have a higher level of fertility awareness. It is suggested that students may be delaying childbearing for social reasons but also for lack of awareness of age-related declines in fertility [18]. In Denmark, Sørensen et al. (2016) found that university college students lack knowledge of the age-related decline, especially in female fertility, overestimate the success rate of IVF treatment, and underestimate the ability to conceive if having unprotected sex at the time of ovulation [19]. To our knowledge, no qualitative studies have explored reflections on the timing of motherhood among women across Denmark, nor within a social media setting.

This study used Facebook to explore women’s reflections on the timing of motherhood, aiming to provide insight into what is important to women when considering when to become mothers.

## Method

### Study design

This study was conducted as a qualitative study using asynchronous online focus groups on the social media platform Facebook. Facebook has shown to be beneficial in recruitment as it was possible to include participants across the country free of charge. Using Facebook as a platform for data collection is particularly helpful with Danish women of reproductive age, as they are frequent users and familiar with this platform [2]. The applied methodological approach and considerations of running online focus groups with women of reproductive age in a social media setting have been discussed thoroughly in Temmesen et al. (2021) [2]. The Consolidated Criteria for Reporting Qualitative Research (COREQ) [21] were used accordingly in the reporting of the present study.

An inductive hermeneutic approach was used. To define our preconceptions, we agreed on a non-pronatalist understanding within the author group, agreeing that every woman should have the opportunity to become mothers if they wish to, with sufficient knowledge to decide *when* or *if* to reproduce. This understanding was used in developing the interview guide and our approach when facilitating the online focus groups.

### Recruitment and setting

**Fig. 1:**
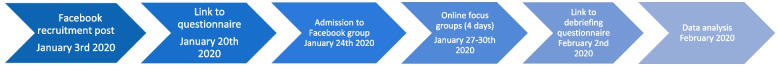
Recruitment and data collection process

Participants were recruited through Facebook using the snowball method within the social network in January 2020. The recruitment post originated from the facilitator’s (1st author) Facebook profile and was shared 287 times. Facebook was chosen as a setting due to recruitment possibilities, popularity, user-friendliness, and the opportunity to create private online groups. The recruitment was purposefully targeted toward women of reproductive age who met the following inclusion criteria: 18–45 years, no children regardless of whether they want to have children in the future or not, the status of being heterosexual, single, or in a relationship, and capable of speaking and writing Danish. A link to information about the study and access to an online questionnaire with sociodemographic questions (age, region of residence, area of residence, educational level, and relationship status) was shared with potential participants. After giving written consent for participation, an invitation link was shared with participants’ personal Facebook profiles, with access to a private Facebook group (Fig. 1). Twenty-six women agreed to participate. Participants were divided into three age groups: *(i)* women aged *18–24* (*n* = 8), *(ii)* women aged *25–34* (*n* = 11), and *(iii)* women aged *35–45* (*n* = 7), and a private Facebook group was created for each age group. Dividing participants into different age groups in this study was based on experience from an online pilot study by Temmesen in 2016, where mixing women of all ages within the reproductive age span did not have the desired intention and caused some participants to feel stigmatized as being either the youngest or the oldest participant. An aim of 8–10 participants in each age group was targeted.

### Data collection

Three asynchronous text-based online focus groups were conducted for three days between January 27th and 29th, 2020, on Facebook. We prepared an interview guide similar for all three age-groups with open-ended questions focusing on three main themes: *(1) Considerations regarding when to have children*,* (2) The influence of others*, and *(3) Age and women’s fertility* (Appendix 1). The 1st author facilitated the focus groups and administered all three Facebook groups. Each day, participants received questions from the facilitator related to one of the three main themes to be discussed in the groups within the next 24 h. The asynchronous approach was chosen to enhance flexibility for the women to participate in the online focus groups whenever it suited them best and to allow greater reflexivity to the questions asked [2]. A week later, participants received an online debriefing questionnaire to anonymously evaluate how they experienced participating in an online focus group. These results are presented in Temmesen et al. (2021) [2].

There was a variety of interactions within each of the age-groups, with a lower level of interactions in the younger group of women (age 18–24 years) compared to the other age-groups. The participants in this group did not as often address specific questions to other participants different as in the different age groups, leading to fewer comments and less lively discussions in this group. The three online focus groups generated ninety-seven full text pages of transcripts (Table 1) written by the participants.

**Table 1:** Interactions within the age-groups

Age-group	Number of interactions	Number of transcribed pages
18–24 years	75	21
25–34 years	195	39
35–45	157	37

The transcripts were copied from Facebook into a text document. Data included Facebook *tagging* (linking a person to a post or a comment by adding his/her profile name), *reaction symbols* (a series of animated emoji reactions), and *emojis* (a small digital image used to express emotions), allowing participants to express non-verbal and emotional communication through web-based language, which contributed to richness in the data [2]. Reaction symbols and emojis were not analyzed independently but presented as a part of the text-based data. Thus, quotes, including emojis, slang language, and original typing, were included and translated from Danish to English after analysis to reduce the risk of losing important nuances. To anonymize participants, identifiable profile names were replaced with pseudonyms, and profile photos were deleted. Data were stored and secured in OPEN (Open Patient Data Explorative Network, University of Southern Denmark).

### Ethical considerations

The Regional Ethical Committee and the Danish Data Protection Agency were notified about the study, but approval was unnecessary for this type of study [22, 23]. The study followed the principles of the Helsinki Declaration [24] and The Danish Code of Conduct for Research Integrity [25], which included that the participants received written information about the study, were informed about the right to withdraw from the study and were ensured confidentiality before giving their informed consent to participation [24, 25].

## Analysis

To enhance the validity of the findings, two authors (CGT and JC) analyzed the transcripts. The themes and subcategories derived from the data were thoroughly discussed and reached by consensus. To enhance transparency, we used quotes to illustrate the statements from the participants. Data were analyzed across age groups, and age and relationship status of each participant was highlighted in the quotes.

Data were analyzed using Malterud’s Systematic Text Condensation, originating from Giorgi’s phenomenological approach, which, according to Malterud, is eligible for all types of qualitative research [26]. Systematic Text Condensation is a descriptive pragmatic analytical approach consisting of four steps: *(1) Overview - from chaos to introductory themes*,* (2) Identification and sorting of meaningful units - from themes to codes*,* (3) Condensation* and *(4) Synthesis* [27]. An example of the analytical process can be found in Table 2.

**Table 2:** Example of the analytical process using systematic text condensation

1. Overview from chaos to introductory themes	2. Identification and sorting of meaningful units - from themes to codes	3. Condensation	4. Synthesis
*Identification of preliminary themes.*	*Extracting meaningful units*,* sorting into codes (decontextualization of data).*	*Dividing codes into categories and subcategories*,* by making condensates.*	*Developing descriptions based on condensates (reconceptualization of data).*
***Fear***	***Achieving pregnancy can be challenging.*** *It took my parents 5 years to get pregnant*,* so there is that fear that when you finally say; now it is now*,* then you can’t….* *Can we have children at all when I am 30?* *Now I fear if*,* what if I’ve waited too long*,* I have not gotten pregnant yet and all the horror scenarios are running around in my head*	***What if it is difficult to get pregnant?*** *Fear of having trouble getting pregnant* *Fear of never being able to achieve pregnancy.* *How will this affect our relationship?*	***What if we can’t have children?*** *Fear of having trouble getting pregnant or*,* in the worst case*,* not being able to have children at all*,* appears as part of women’s consciousness when they think about having a child in the future. The fear is accompanied by thoughts about how it will affect their mental health if they must go through fertility treatment*,* as well as they worry about how this may affect their relationships.*

**Table 3:** Characteristics of participants in the online focus groups

PARTICIPANTS (*n* = 26) Name (pseudonym)	AGE (range 18–43 years)	EDUCATIONAL LEVEL*	RELATIONSHIP STATUS	REGION OF RESIDENCE
***Anna***	39	Medium	Single	Southern Denmark
***Amelia***	31	Medium	Single	Southern Denmark
***Ava***	31	Medium	Single	Zealand
***Beatrice***	32	Higher	Single	Capital
***Catherine***	30	Higher	In a relationship, cohabiting	North
***Charlotte***	28	Higher	In a relationship, not cohabiting	Capital
***Emily***	43	Medium	Single	Capital
***Hannah***	32	Medium	In a relationship, cohabiting	Capital
***Helen***	35	Higher	In a relationship, not cohabiting	Zealand
***Julia***	23	Medium	In a relationship, not cohabiting	Capital
***Josephine***	32	Medium	In a relationship, not cohabiting	Capital
***Lauren***	38	Higher	Single	Mid Jutland
***Lily***	27	Higher	In a relationship, cohabiting	Capital
***Megan***	23	Medium	In a relationship, cohabiting	Zealand
***Mia***	25	Medium	In a relationship, cohabiting	Zealand
***Olivia***	35	Shorter	In a relationship, cohabiting	Capital
***Sarah***	25	Other	Single	Mid Jutland
***Sophie***	33	Higher	In a relationship, cohabiting	Southern Denmark
***Susan***	19	Primary	In a relationship, cohabiting	Zealand
***Tessa***	29	Medium	Single	Southern Denmark
***Tori***	36	Higher	Single	Southern Denmark
***Tracy***	38	Higher	In a relationship, cohabiting	Southern Denmark
***Vanessa***	26	Higher	Married	Capital
***Vera***	18	High school	In a relationship, not cohabiting	Southern Denmark
***Veronica***	24	Medium	Married	Capital
***Victoria***	20	High school	In a relationship, cohabiting	Capital

* Educational levels: Primary school, High school, Shorter education (2–3 years), Medium higher education (3–4½ years), Higher education (5–6 years) and other

## Findings

The findings represent 26 women, each of whom had their own unique reflections on the timing of motherhood. The characteristics of the participants are described in Table 3.

Three main themes, each with four subthemes, emerged from the data: (1) Life before parenthood, (2) Planning for a child and (3) Life without children.

## Life before parenthood

The first main theme, *‘Life before parenthood’*, consisted of four subthemes, which addressed the women’s thoughts of having children prior to actual planning for a child: (1) *Fulfilling other dreams*, (2) *Comparison to others*, (3) *Loss of freedom* and (4) *Feeling the pressure*.

### Fulfilling other dreams

Most of the women express having other dreams, not directly related to having a child, that they want to fulfill before having children:

***Beatrice ****(age 32*,* single)*: *‘I’m very much looking forward to having children one day*,* but I still don’t feel quite ready to compromise my lifestyle and*,* among other things*,* my many journeys´.*

Completing an education, pursuing a career, and having a spontaneous life with the possibility of traveling and enjoying being just two in a relationship are portrayed as being important factors to achieve before starting a family.

### Comparison to others

When asked if they’ve ever imagined *when* to have children, several of the women tend to compare themselves with their immediate family and female friends and colleagues. In particular, concerning the biological conditions of becoming a mother, the woman’s own mother, sister or aunt is often used as a reference for comparison:

***Megan ****(age 23*,* in a relationship*,* cohabiting): ‘I do not feel that I am in a hurry to have children yet*,* since most of my own family has been 30 years or older before having their first child. It gives me at least seven more years to run on*  ’.

Regarding the social conditions surrounding future motherhood, friends and colleagues are often used as a reference for comparison. Thus, women compare their own fertile situation with their immediate family and the social norms that exist among friends, colleagues or in the communities in which they live, work, or socialize.

### Loss of freedom

Some women express fear of losing freedom when establishing a family:

***Hannah ****(age 32*,* in a relationship*,* cohabiting): ‘…At times*,* I feel like I’m ready for it*,* but I also love being “free”. I enjoy just being the two of us too much to have children now’.*

Thus, having a child is expressed as having less freedom and a fear of missing out on something else, which is why it is important to have a sense of freedom before they feel ready to plan for a future family.

### Feeling the pressure

Several of the women, especially those in the age group 25–34 and 35–45 years, describe how their closest family members express joy and expectations when they talk about starting a family. On the opposite, if the women do not have a partner or if they express ambivalence toward motherhood, their families are concerned about whether they will have children at all. Sometimes, this pressure from family and friends is perceived as positive and well-intentioned care, but for some of the women, this form of attention is experienced as a negative pressure:

***Lily ****(age 27*,* in a relationship*,* cohabiting): ‘My family has behaved badly since I found my current boyfriend*,* and over the past year*,* it has escalated completely crazy. When we attend family gatherings*,* my family mentions several times whether we should have children soon*,* and my grandmother says*,* “When should I have great-grandchildren? ”… it frustrates me*,* especially because we are trying to conceive (…)’.*

In addition, women from all age groups describe negative pressure from friends and colleagues when discussing when to have children:

***Sophie ****(age 33*,* in a relationship*,* cohabiting): ‘I have felt and still feel an enormous amount of pressure from friends and colleagues (…) Many have asked if my boyfriend and I are going to have children soon. I’ve been very quick to reject it*,* as I would rather decide for myself when to have children - and because we are going to have children for OUR sake and not for the sake of others and the expectations of others’.*

In particular, the younger women (age group 18–24 years) describe how they face prejudice and feel that others see their opportunities to succeed later in life as less if they indicate that they want to have a child in their early or mid-20s:

***Susan ****(age 19*,* in a relationship*,* cohabiting): ‘I think everyone has an opinion on when you should or should not have children (…) It is natural for me to listen to the arguments they come up with’.*

The younger women describe how societal stigmatization of young mothers makes them doubt whether it is better to wait to have children at a time when it would be more socially acceptable to have children:

***Vanessa ****(age 26*,* married): ‘I had a friend who had her first child when she was 26*,* and there was a wide consensus in the circle of friends that it was very early… I think such things made me think that I should rather wait a bit’.*

On the opposite, in the oldest age group (35–45 years), a woman describes how society expects her to have a desire for motherhood:

***Tori ****(age 36*,* single): ‘They do not understand that you have no desire and need at all. Especially those with children clearly feel that I’m missing out on something*,* they almost think it should be mandatory for all women to have children…’.*

Women who, for various reasons, have chosen not to have children experience that family, friends, and colleagues do not understand their choice:

***Lauren ****(age 38*,* single): ‘… others are trying to persuade me to have children. Especially my colleagues (…)”. Do not cheat yourself because it is the most amazing thing” and at the same time they try to convince me that I will truly regret it later if I* do not….”.

## To plan for a child

The second main theme, *‘To plan for a child’*, addresses the perspectives of planning for a child and consists of four subthemes: (1) Wishing for a child, (2) Creating optimal conditions, and (3) Insecurities towards motherhood, *and (4) The partner as gatekeeper.*

### Wishing for a child

Several women describe how they have dreamt of having children since childhood or adolescence. This dream often addresses a certain, but not specific, age where they imagine that it will be optimal to have children:

***Tessa ****(age 29*, *single): ‘I have always wanted to have children and that is what I “dreamed about’. As a young girl*, *I imagined having my first child when I was 23. Now I turn 30 in February and there are still no children on my way* * It has been a great sadness for me for a while that I have not had children yet*, *but now I’m more concerned about finding love…’.*

As described above, several of the women feel that their plan for having a child has been unsuccessful, either because they have not met the right partner to establish a family with or because their partner is not ready to have children or because something else has come in the way, such as education or career. At the same time, Tessa’s statement outlines the struggle single women may face when longing for motherhood while having a strong desire to find the right partner with whom to share parenthood. Furthermore, the women describe how some of them are hesitant to talk to their friends, relatives, or their general practitioner about considerations regarding when or if to have children:

***Hannah ****(age 32*,* in a relationship*,* cohabiting): ‘I don’t know why I do not talk about it with my friends about it*,* it may be a little too personal… at least for now (…) I don’t quite know why I think it is such a personal topic of conversation (…) I’m not going to talk to my doctor about children until I get pregnant or have problems. I don’t think she has anything to do with me and my boyfriend’s thoughts on having children’.*

Thus, for some women, thoughts on timing of motherhood are a personal matter, which they do not feel ready to share with friends, family or health professionals.

### Creating optimal conditions

When the women talk specifically about planning motherhood, several prerequisites to be able to create the best possible conditions for a child are considered essential:

***Victoria****(age 20*,* in a relationship*,* cohabiting): ‘Before we bring children into the world*,* I truly want us to have a stable income and a place to live where there is room for family growth’.*

Thus, it is considered important to have completed an education, to have a family-friendly home and financial security to be able to care for children.

### Insecurities towards motherhood

Some women express doubts as to whether they will be able to fulfill the role of motherhood. Women who consider themselves vulnerable or have experienced stress or depression in the past express concerns about how motherhood will affect their mental health:

***Catherine ****(age 30*,* in a relationship*,* cohabiting): ‘…The women in my family tend to have birth depression and to have subsequent psychological challenges for the rest of their lives*,* and it scares me (…) I have no idea if birth depression is inherited*,* but both my mother*,* grandmother and great-grandmother experienced it’.*

Another woman describes a fear of not being able to love the baby the way she feels a mother should love her child:

***Charlotte ****(age 28*,* in a relationship*,* not cohabiting): ‘I’m scared to death that I will not love my kids immediately. I have never thought children were particularly sweet’.*

Thus, some women have concerns about their role as a mother, which has an impact on whether they dare to throw themselves into motherhood.

### The partner as gatekeeper

Several of the women in a relationship state that they feel ready to have a child, but their partner is not yet ready to become a father. Some women express doubts about whether their partner wants children at all, especially if the partner previously stated that he does not envision having children in the future. For this reason, some women explain how they are careful not to push the partner too much:

***Julia ****(age 23*,* in a relationship*,* not cohabiting): ‘Honestly*,* I’m careful of what I say about having kids to my boyfriend. He knows very well how sick I am [*for having a child*]*,* and he is tired of hearing about it - so as not to give him more pressure than he already has*,* I make sure to talk to someone else about it’.*

This illustrates how some women are waiting for their partner to become ready for the parenting role and imagine that the partner’s wishes for a future parenthood will mature. It is described as stressful when the urge to become a mother is present, if the partner is missing or if the partner does not immediately want a child.

## A life without children

The third main theme, *‘A life without children’*, consisted of four subthemes, which addressed different aspects of a possible future life without children: (1) *What if we can’t have children?* (2) *Not longing for motherhood*, (3) *Fear of loneliness* and (4) *Finding new strategies*.

### What if we can’t have children?

Fear of not being able to achieve pregnancy if parenthood is postponed, is part of some women’s consciousness as they think about their chances of having children in the future:

***Lily ****(age 27*,* in a relationship*,* cohabiting): ‘Now I fear what if I have waited too long*,* I have not gotten pregnant yet and all the horror scenarios run around my head* *’.*

Two younger women discuss the fear of facing fertility challenges or not being able to achieve pregnancy at all:

***Veronica ****(age 25*,* married): ‘* * It took my parents 5 years to become pregnant*,* so there is also the fear that when you finally say; Now it is time*,* you cannot. I think there are many things to consider*  .

***Julia ****(age 23*,* in a relationship*,* not cohabiting): ‘I truly do have a fear that it will take way too long or at worst*,* not to succeed at all. It is also one of the reasons why I would like to get started early*,* so that it certainly does not end up being the “fault” of age and thus something could have been done.*

***Veronica ****(age 25*,* married): ‘Exactly*,* I am influenced by the fact that my parents were 25 and 24 years old when they started and I am*,* as I said*,* 25 years now’.*

Fear of experiencing fertility challenges is accompanied by thoughts about how this can affect the relationship:

***Olivia ****(age 35*,* in a relationship*,* cohabiting): ‘I have a fear of what if we (I) cannot [*become pregnant*] at all? (…) However*,* if you reach a point where you decide to try*,* you run the risk of becoming one of the couples where it becomes an all-consuming project where you must bang on certain times and at special temperatures?!* *What strain does it put on our relationship if we decide to try …?’.*

### Not longing for motherhood

Women who do not imagine themselves as mothers have various reasons for this, for example, if they had never experienced a longing for motherhood:

***Emily ****(age 43*,* single): ‘I have never had the need or longing to start a family and become a mother. Considered it in the early 20 s*,* but there was so much else that occupied me at that time…’.*

Others describe that they are doubting whether they will be able to fulfill the parenting role, which has led them to opt out of motherhood. However, some women feel that they have not consciously made a choice about not wanting children but rather that this is just how life ended up:

***Tori ****(age 36*,* single): ‘I have not deliberately opted out of having children*,* but I have never felt a need to become a mother - and since I have been single since my early 20 s*,* it just has not been in the cards’.*

### Fear of loneliness

When facing a possible future life without children, some of the women describe a fear of loneliness when reaching old age:

***Tori ****(age 36*,* single): ‘My only “concern” about not having children is whether I get lonely when I get older (…) I sometimes think if I will feel alone in 20 years when my parents are old/gone*, *and I do not have my own family. Where for example*,* will it feel natural to go for Christmas and such’.*

A life without children also means facing a life without grandchildren:

***Emily ****(age 43*, *single): ‘Half a year ago*, *I came to the thought that when I opted out of children*, *I will not have grandchildren – that hurt! I’m sure I would have loved to be a grandmother* *’.*

Thus, a life without children is not only about the absence of children or opting out of motherhood but involves and has significance for other life stages.

### Finding new strategies

If the women envision themselves being unable to have biological children, they are positive toward finding alternative strategies such as oocyte donation to achieve pregnancy:

***Catherine ****(age 30*,* in a relationship*,* cohabiting): ‘If it is not possible with my eggs*,* then fortunately there is also the help of retrieving there: ) (…) For me it is just before it is an advantage if it is not my genes if it makes sense? Then*,* I do not have to worry about whether my child inherits the mental challenges that have been in my family for several generations. :)’.*

Adoption is also mentioned as an alternative strategy toward having a life with children:

***Megan ****(age 23*,* in a relationship*,* cohabiting): ‘I*,* myself*,* am reasonably clear that if I/we are unable to have biological children*,* I/we would like to adopt’.*

When the women articulate the possibility that they may not have their biological children, whether they consider themselves involuntarily or voluntarily childless, they find new strategies to have children in their lives. For example, having a close connection to friends and family’s children, a pet, or as a bonus mother for a potential partner’s biological children:

***Tori ****(age 36*,* single): ‘I have wonderful children around me in the family and in my circle of friends*,* and it satisfies me greatly (that*,* and so does my cat*,* which is*,* after all*,* a form of pseudo child: D) (…) I always say that there will probably be available men with sweet children*,* where you can become a bonus mother*,* when the marital crises hit* * Then*,* you can have the relationship and the mother role*,* if you need it‘.*

Thus, the women are creating alternative ways of having children in their lives and emphasizing having strong relationships with children of relatives and friends:

***Lauren ****(age 38*,* single): ‘I am desperately trying to hang on to my nephews and niece so they can take care of me when I get old*  …*’.*

## Discussion

By selecting women with no children, regardless of whether they want children in the future or not, we gained important knowledge of the reflections on timing of motherhood from women of reproductive age across Denmark.

In this study exploring reflections on the timing of motherhood among reproductive aged women with no children we found that the decision to have a child is complex and does not only depend on the woman’s desires, choices, and actions alone. We discovered what we identify as *external* and *internal conditions* that women in this study describe ideally should be met for them to feel ready to have children. Having a partner who is willing to share parenthood, having pursued an education, being financially secure, and having a suitable residence play a significant role in *external conditions* when women consider the timing of motherhood. In a Swedish study, Eriksson et al. (2012) describe this as an ‘important prerequisite’ for having children [28]. Söderberg et al. (2012) describe this as creating ‘a perfect life’, to be understood in this context that when women plan their lives regarding having children, they strive to plan these conditions in a certain order to create the best possible conditions for a life with children [29], a finding that is supported in a Danish study by Birch Petersen et al. (2016) [9]. Additionally, we identified *internal conditions* that women in this study described ideally should be present within themselves to consider having children, e.g., to have felt a sense of readiness to become a mother, feeling a desire for motherhood, having confidence in one’s own parenting skills and willingness to give up other things in life, such as traveling, enjoying being just two in a relationship, etc., before having a child. Eriksson et al. (2013) describe this as a ‘consequence of competing priorities’ when young men and women are going through adulthood, and planning to have children is one of them [30].

Most of the women in this study had dreamt of motherhood since a very young age, and several had imagined that they would be mothers at a certain, but not clearly defined, age. For the youngest age group (18–25 years), the women envision an ideal age when they see themselves as mothers in the future, ideally when the external and internal conditions have been fulfilled. Most women from the age group 26–35 years and age group 36–45 felt that they already had exceeded the imagined age, where they had expected to have become mothers earlier in life. Still, it stated that they had not met the external or internal conditions they felt were necessary to pursue motherhood. In Eriksson et al. (2012), this ideal undefined age for having children is described as a balance between ‘not being too young or too old’ [28]. Across all three age groups, a few women were unsure whether they would have children in the future, expressed as an ambivalence toward motherhood. Some of these women doubted whether they could provide the optimal maternal care for a child in a way they envisioned motherhood ideally should be. In an Australian study, Maher & Saugeres (2007) support this finding, as they find women without children to have a very idealized and traditional view of motherhood. In contrast, they find women with children to have a far more pragmatic and less idealized view of motherhood [31]. Birch Petersen et al. (2016) describe this as an idealized perception of perfect mothering with unrealistically high aims and an uncompromising approach in the eagerness to become a ‘perfect mother’ [9].

Most of the women in this study described an expectation of having the same reproductive opportunities or limitations as their biological female relatives. For example, an expectation of being able to have children at mid- or late thirties if a mother, sister, or aunt had children at advanced maternal age, or the opposite, expecting to be challenged on their fertility if someone in their immediate family has experienced fertility problems. This indicates that women biologically identify themselves with their immediate female family members and compare their fertility conditions with their female relatives. This is an important finding since comparison to relatives may pose a pitfall for some women, as women’s fertility conditions are individual and can vary considerably. Eriksson et al. find that it is common for women to relate to the age of when their parents had children [28], but to our knowledge, our finding of women’s expectations of having the same biological fertility conditions as their relatives is new. This indicates that women lack knowledge about the impact of age on fertility, and this finding stresses the importance of raising awareness on how age affect women’s reproductive opportunities, including educating women not to assume they have the same reproductive opportunities as their female relatives. It also highlights the importance of reproductive counseling to address individual reproductive conditions, and the importance of disseminating knowledge of age-related fertility challenges.

Regarding the social norms of the timing of motherhood, the women in this study compared themselves with their female friends and colleagues. Friends’ and colleagues’ opinions about when it is the right time or not to have children contributed to their perception of the ideal time to have children, indicating that social norms play a significant role in women’s timing of motherhood. The impact of social norms surrounding childbearing is supported in a British study by Lavender et al. (2015), who describes how society frames the ‘acceptable’ age to have children, for example, in the way, media plays a role in women having children later in life when portraying older celebrity mothers positively. Furthermore, Lavender et al. (2015) describe that younger women between 25 and 34 years more often have multiple sources of social comparisons, such as media, friends, mothers, or colleagues. In contrast, women above age 35 years have a clear reference group of friends of a similar age with children [6].

In this study, we identified that women, especially in the age groups 26–34 and 35–45 years, felt pressure from family and friends, who often commented on their reproductive situation because they had not yet had a child. This pressure could be experienced as positive, motivating, and caring, or the opposite, as negative, demanding and interfering in the women’s reproductive considerations. Women in a stable relationship with a partner who also wanted to start a family considered the pressure from family and friends positive, filled with mutual joy and expectations for the future. Women who were longing for motherhood but were not able to achieve it, either because of the lack of a partner or because of disagreement about desires for future parenting with an existing partner, felt that this pressure contributed negatively to their reproductive situation and represented unfulfilled dreams from the women’s families and, in some cases, within the women themselves. Women, who for various reasons opted out of having children, experienced being met with a lack of understanding from friends and family. Maher & Saugeres (2007) describe that because motherhood is often constructed as being a highly desirable and natural course in life for many women, women who have made a decision not to have children or who have difficulties deciding whether to have children are portrayed as being abnormal or having emotional problems [31], adding to a stigmatization of voluntarily childless women.

Younger women (age-group 18–25 years) felt stigmatized by friends and family, if they considered having a child within this age span. This finding indicates that Danish women experience a social norm for ‘the right time’ of having children, neither being ‘too young’ or ‘too old’.

Some women described a longing for motherhood, but the partner or the lack of a partner stood in the way of the women fulfilling their wishes for a future motherhood. When men postpone fatherhood, it indirectly impacts their partners. The male perspective is not included in the current study; however, it is conceivable that similar considerations regarding the timing of parenthood apply to both men and women. Men and women must be aligned when planning future parenthood; therefore, more is needed to consider only women’s perspectives. Women in relationships described how they were hesitant to discuss whether to have children with their partner if the partner had expressed ambivalence toward having children. They fear pushing their partner into parenthood or having to end the relationship if their wishes for a future with or without children were too different. This leaves childless women in a dilemma about whether the love of the partner or the desire to have a child determines their future options for motherhood. In a Danish study, Werner et al. (2021) describe how women who have chosen solo motherhood (being a single mother by choice) have pursued pregnancy without a partner because they felt the time was running out due to increasing age and decreasing fertility [32].

Not all women were certain that they would become mothers in the future, so thoughts about a life without children were inevitable. The women described how they were looking for new strategies for having children. For women who longed for motherhood, these new strategies included being positive toward oocyte donation or adoption. Women who were ambivalent toward motherhood or had accepted that they probably would not have children stressed the importance of having a strong relationship with children within the family or being involved in the lives of children of close friends. When facing a senior life without grandchildren, a concern was expressed for several of the women who were ambivalent toward motherhood.

Some women described how they had never felt a longing for motherhood and that they were having a hard time imagining becoming mothers. We consider it an important finding that these women nevertheless volunteered to participate in this study, which indicates that women who, for various reasons, are not planning to have children yet want a voice in the debate on the timing of motherhood.

Some participants stated that they consider it a private matter to discuss when or if to have children and expressed that they were hesitant to discuss the timing of motherhood with friends, family, colleagues or their general practitioner. This indicates that health professionals need to consider how they communicate health preventive services for women of reproductive age, just as we cannot take for granted that women want to disclose their reproductive choices at any given time. This finding stresses the importance of including women when developing new initiatives within the reproductive field. Nevertheless, the women in this study showed willingness to connect and discuss motherhood reflections with strangers, even disclose vulnerable thoughts on motherhood, which indicates that the online universe offers a form of confidentiality that is not necessarily given outside the online environment. Tates et al. (2009) found that online participation increases the level of self-disclosure compared to traditional focus groups. Further, that the perceived privacy of the online environment causes the participants to give honest and reflected answers.

Asynchronous online focus groups have the advantage that socially or geographically isolated or introvert people can participate to their convenience without time-pressure [33]. As some women in this study expressed reluctance to discuss family plans with friends and family, but nevertheless were willing to participate in the discussions online, a relatively anonymous online environment with peers may be encouraging for some women. Thus, discussing considerations of motherhood online with other women of reproductive age has the potential to support women in their reflections of when and if they are ready to pursue motherhood.

### Strengths and limitations

The participants in this study represent a group of Danish women who were willing to discuss the timing of motherhood with other women of reproductive age in an online setting. This study, from the perspectives of the women themselves, offers an in-depth understanding of Danish women’s reflections on the timing of motherhood. Recruiting women without children made it possible to capture the essence of considerations of the timing of motherhood from women who may or may not become mothers in the future.

The strengths and limitations of the applied online method have been elaborated in Temmesen et al. (2021) [2].

We recruited women who defined themselves as heterosexuals, as we hypothesized that homosexual women may have specific circumstances surrounding timing of motherhood that differ from heterosexual women, including considerations regarding shared motherhood, the need for a sperm donor, and limited opportunities to access fertility treatment for LGBTQ + individuals in some countries. However, we acknowledge that there can be similarities between homosexual and heterosexual women regarding to considerations of timing of motherhood.

Most of the women shared a common characteristic in the form of a high educational level (medium or higher education), although we did not deliberately aim for this. This can be considered a selection bias, but we aimed to recruit women who wanted to discuss the timing of motherhood, and Danish women, in general, have a high educational level. As compensation for a rather homogeneous sample of participants regarding educational level, we recruited women with various backgrounds in terms of geographical area of living and relationship status, and we included women from different age groups, representing the age span for Danish women of reproductive age. However, the youngest group (18–24 years) in this study had a lower level of interactions and provided fewer general comments than the other age-groups. This might be because this age group is having difficulties in relating to becoming mothers, whereas the thought of motherhood can be experienced more relevant for women in the other age groups.

Despite the limitations, the findings from this study can be transferred to women of reproductive age in similar contexts.

Although we recognize that reproductive considerations involve both men and women, this study focuses exclusively on women’s reflections on the timing of motherhood.

## Conclusion

This study provides insight into reflections on the timing of motherhood among Danish women of reproductive age. Several internal and external considerations influence *when* and *if* women feel ready to pursue motherhood. Women tend to compare themselves with their female relatives regarding their biological capacity for having children and compare themselves with their friends and colleagues regarding the social norms surrounding motherhood. Furthermore, women of reproductive age often experience a pressure from family, friends, and colleagues about when to have children, which can be experienced as either positive or negative. Women in this study were positive towards discussing timing of motherhood with other women of reproductive age on social media.

### Implications for practice

Online communities like Facebook groups have the potential to provide a gathering place for women of reproductive age and support women’s reflections on pursuing motherhood. Thus, online groups should be considered when implementing new health initiatives for women of reproductive age. Further research, particularly focusing on men’s reflections on the timing of fatherhood and men’s experiences of discussing timing of fatherhood in online communities, should be considered.

## Supplementary Information

Supplementary Material 1.

## Data Availability

The datasets used and analyzed during the current study are available from the corresponding author on reasonable request.
