# Supplementary material for: Reflections on timing of motherhood - a qualitative online study with women of reproductive age
Source: BMC Womens Health. 2024 Nov 5;24:589. doi: 10.1186/s12905-024-03409-0 (PMC11536869; doi:10.1186/s12905-024-03409-0)
Supplement: Supplementary file 1 — Supplementary Material 1. [file 12905_2024_3409_MOESM1_ESM.docx]

**Appendix 1: Interview Guide ‘When will you become a mother?’ (translated from Danish to English for article purposes only).**

**1st Pre-briefing Post, January 24, 2020:**

Dear Participants,

Thank you very much for your willingness to participate in the online focus group ‘When will you become a mother?’, which is part of a Ph.D. project at the University of Southern Denmark. The online focus group will take place from Monday, January 27 to Thursday, January 30, 2020 (week 5). The purpose of the online focus group is to gain more knowledge about Danish women’s considerations regarding when they want to become mothers. You will receive a question/prompt in the group daily, which you are asked to answer and discuss with each other over the next 24 hours. You are, of course, welcome to go back later in the process and elaborate on your answer to a previous topic if you think of something new.

The basic idea behind an online focus group is that you discuss your considerations with each other. There are no right or wrong answers, as it is precisely your different thoughts and considerations that are interesting. Therefore, it is not necessarily the intention that there should be agreement in the group, but rather to get your different viewpoints on the subject. You are welcome to ask each other to clarify questions, and it is also perfectly fine if you happen to move a little away from the original topic. I will not directly participate in the discussions, but you will experience that I may ask clarifying questions along the way.

Since we are not sitting across from each other physically and lack body language, you are welcome to indicate that you have seen the post by commenting. If you do not know what to answer or do not want to answer, just write that. You are free to use emojis/photos and the like as part of your communication in the group.

Since the online focus group is part of a research project, I ask you to respect that it is only you as participants and the researchers behind the project who have access to the content in the group. Although it is intended that the knowledge that emerges in the online focus group should be disseminated, it is important that we can all count on confidentiality about what is written in the group so everyone feels that we can communicate freely. Since the online focus group takes place on Facebook, the research is subject to Facebook’s data policy, as you know it when you use Facebook as social media in general. You can read more about Facebook’s data policy here [Facebook Data Policy](https://www.facebook.com/privacy/explanation).

When the online focus group is completed, the group will be deleted after one month. Data will subsequently be stored on the University of Southern Denmark’s server, and your name and profile photo will all appear in anonymized form. Your participation is voluntary, and you can withdraw from the project at any time. If you regret your participation, please contact me as soon as possible at ctemmesen@health.sdu.dk. Please let me know if there is anything you want elaborated.

Best regards,

Project Manager/Ph.D. student Camilla Gry Temmesen

**2nd Pre-briefing Post, January 26, 2020:**

Dear Participants,

On Monday, January 27, 2020, the online focus group ‘When do you want to become a mother?’ starts and continues until Thursday, January 30, 2020. I am pleased that you want to contribute to this research project, and I am really looking forward to hearing your thoughts and considerations about motherhood. Remember that the success of the group depends on your input and how much you interact with each other, so do not hesitate to comment, ask each other questions, or elaborate on your thoughts along the way. You are welcome to use text, photos, emojis, videos, or the like to support your thoughts and considerations.

Since we are not going to meet each other physically, I ask you to tell a little about yourself to the other participants as a short presentation round. Just share what you want, e.g., your age, relationship status, job/education, in which part of the country you live, or similar. You are welcome to do it right away, but you can also wait until Monday if it suits you better. Remember to indicate that you have seen a post by liking or commenting on the post.

Best regards,

Project Manager/Ph.D. student Camilla Gry Temmesen

**Day 1, Monday, January 27, 2020:**

Dear Group,

Welcome to day 1 of the online focus group. Today, I would initially ask you to tell a little about yourself to the other participants as a small presentation round, if you have not already done this. Just share what you want, for example, your age, relationship status, job/education, in which part of the country you live, or similar.

The question that I would ask you to discuss in the group is: What are your considerations on when you want to have children?

If it is a bit difficult to get started, the following support questions may set your thoughts in motion, but these support questions should only be considered as inspiration. Remember, you should discuss your considerations with each other, so you are very welcome to ask or comment on what is being written in the thread. NOTE: Write your comments in the thread attached to this post, so it is easier to find again.

Support Questions:

- What age do you imagine having your first child?
- Why is this age/this time ideal or decisive for you?
- What priorities do you have that you would like to fulfill BEFORE you have children?
- What holds you back from having a child, for example, within the next year?
- What speaks for having a child, for example, within the next year?
- Does the thought of starting too late to have children occupy your thoughts?
- Does the thought of regretting your choice (for example, about waiting to have children/not having children/at all to have children) occupy your thoughts?
- If you have chosen not to have children, what lies behind your choice?

**Day 2, Tuesday, January 28, 2020:**

Dear Group,

Today we have reached day 2 in the online focus group. Thanks for the many interesting discussions on yesterday’s topic. You are welcome to continue to comment in the thread. Today, I would ask you to discuss the question: How do you experience that the outside world affects your considerations about having children?

Support Questions:

- Please define what you mean by the outside world. Is it your potential partner, family (parents, siblings, grandparents), friends/girlfriends, politicians, media, or other?
- Do you experience that the outside world affects you negatively when it comes to having children?
- Do you experience that the outside world affects you positively when it comes to having children?
- Does it make you want to have children earlier or later?
- Have you told your outside world about your considerations or choices?
- Who is the closest person you talk to about your considerations regarding having children? (for example, potential partner, parents, siblings, friends/girlfriends, health personnel, or others?)
- Is it difficult to talk to others about it? Why/why not?

**Day 3, Wednesday, January 29, 2020:**

Dear Group,

We have reached day 3 in the online focus group. Today, I would ask you to look closely at the graph:

Explanation: The graph shows women’s monthly probability of achieving pregnancy, leading to the birth of a living child, and as you can see, this probability decreases with the woman’s increasing age. A healthy 20-year-old woman has a 33% chance of getting pregnant (per cycle). As a 30-year-old, this probability is halved to 17%, rapidly falling to 8% as a 37-year-old, and as a 45-year-old, the chance of achieving the birth of a living child is down to 0.5% per cycle.

What do you think about this information? Write what immediately comes to mind.

Support Questions:

- When you get information like this, what does it make you think?
- Does this information affect your considerations about when you want to have children? If so - how?
- Does this correspond to the knowledge you already have about women’s fertility and age - or are you surprised by this?
- Had you imagined that the probability of getting pregnant was higher with increasing age than the graph shows?
- Had you imagined that the probability of getting pregnant was lower with increasing age than the graph shows?
